# The observation of anterior segment in children with an R124L mutation corneal dystrophy by anterior segment optical coherence tomography and in vivo confocal microscopy

**DOI:** 10.3389/fmed.2022.991204

**Published:** 2022-10-28

**Authors:** Mengjun Fu, Jing Zhao, Haorun Zhang, Rui Wang, Xingtao Zhou

**Affiliations:** ^1^Weifang Eye Hospital, Weifang, China; ^2^State Key Laboratory of Ophthalmology, Optometry and Visual Science, Eye Hospital of Wenzhou Medical University, Wenzhou, China; ^3^National Clinical Research Center for Ocular Diseases, Eye Hospital of Wenzhou Medical University, Wenzhou, China; ^4^Eye Institute and Department of Ophthalmology, Eye and ENT Hospital, Fudan University, Shanghai, China; ^5^National Health Commission Key Laboratory of Myopia, Fudan University, Shanghai, China; ^6^Key Laboratory of Myopia, Chinese Academy of Medical Sciences, Shanghai, China; ^7^Shanghai Research Center of Ophthalmology and Optometry, Shanghai, China

**Keywords:** corneal dystrophy, R124L mutation, anterior segment OCT, confocal microscopy, children

## Abstract

**Purpose:**

To evaluate the anterior segment in children with an R124L mutation corneal dystrophy (CD) using anterior segment optical coherence tomography (AS-OCT) and in vivo confocal microscopy (IVCM).

**Methods:**

We investigated a family with prevalent CD and an R124L mutation; 59 individuals (14 patients; 6 male and 8 female, aged 2–69 years, 6 children, 2:4 male: female ratio) from four generations were included. We observed corneal lesions through ophthalmologic examinations, AS-OCT, and IVCM. The mean follow-up was 4.60 ± 3.91 years.

**Results:**

The mean age for childhood CD onset was 0.90 ± 0.61 years. An Avelino DNA test revealed a heterozygous R124L mutation. Clinical manifestations included recurrent photophobia, tearing, and a foreign body sensation. Recurrence frequency decreased with age. Slit lamp microscopy revealed a rough corneal epithelium. The anterior matrix under the corneal epithelium and the anterior elastic layer were scattered with gray and white opacity. From onset to follow-up, the children’s visual acuity decreased from 0.34 ± 0.12 to 0.55 ± 0.17 LogMAR units. AS-OCT showed uneven corneal epithelial thickness. The Bowman’s layer was replaced by abnormal substances in the anterior segment. Corneal deposits became increasingly thicker; the average thickness at the last follow-up was 102.78 ± 10.13 μm. IVCM revealed uneven and reflective signals in the corneal upper cortex and subepithelium, with unclear boundaries and a loss of normal cell morphology.

**Conclusion:**

We report an early age of onset in a family with prevalent CD due to R124L mutations. AS-OCT is a convenient, quick, and non-contact tool for screening and monitoring the pathological process of CD.

## Introduction

Corneal dystrophy (CD) is a general term for a series of primary, binocular, and blinding corneal diseases related to family inheritance (1). CD caused by an R124L mutation is an anterior CD with autosomal dominant inheritance (2). Generally, the onset of CD occurs early and progresses rapidly. In later stages, corneal spottiness is mapped and gradually expands, and fusion leads to vision loss and blindness. Phototherapeutic keratectomy (PTK) or corneal transplantation keratoplasty is often required (3). Early corneal opacity in children may lead to deprivation amblyopia, which has a long disease course and is prone to recurrence. The examination and treatment of children is sometimes more difficult to conduct than in adults. Therefore, more attention should be paid to early screening and monitoring with respect to CD in children. This study reports a family with prevalent CD and a history of consanguineous marriage. Children with the disease were evaluated *via* an Avelino DNA test and their clinical symptoms were evaluated as well. The anterior segment was observed using anterior segment optical coherence tomography (AS-OCT) and in vivo confocal microscopy (IVCM).

## Materials and methods

### Study population and ethics approval

This study was conducted in accordance with the requirements of the Weifang Eye Hospital Review Ethics Committee as well as with the principles of the Declaration of Helsinki. All clinical examinations and genetic analyses were conducted with written informed consent from the participants and their families.

**FIGURE 1:**
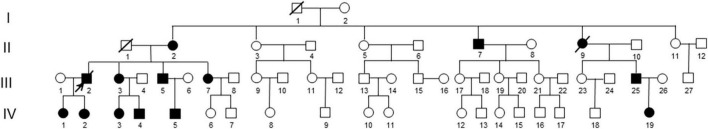
Lineage of a family with prevalent CD caused by an R124L mutation (I1 and I2 were inbred, IV: 2 was 2 years of age).

A family with a history of incest in China was selected for evaluation (Figure 1). A total of 59 individuals (14 patients and 45 healthy persons) from four generations were included in this investigation. This family was entirely of Han Chinese ancestry. There were six children among the 14 patients (male: female ratio, 2:4). The youngest patient was 2 years old.

### Methods

The medical history of all participants was recorded, including the age of onset, clinical symptoms at onset, and visual acuity, slit-lamp microscopy, fundus examination, and other routine ophthalmological examination findings. An Avelino DNA test was performed on children with the disease, and anterior segment photography, AS-OCT, and IVCM were performed during the follow-up period. The mean follow-up time was 4.60 ± 3.91 years (1–11 years).

### Statistical analysis

Statistical Package for the Social Sciences (SPSS) software (version 25.0, Chicago, IL, USA) was used to conduct all statistical analyses. Changes in the children’s visual acuity and OCT sediment thickness were expressed as means ± standard deviations.

## Results

### Clinical parameters

**FIGURE 2:**
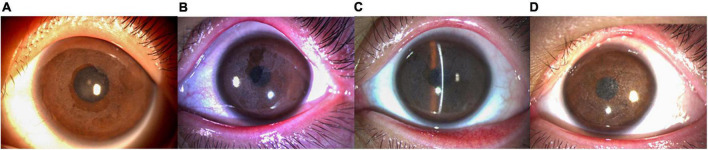
Anterior segment photography (X16, Topcon, Tokyo, Japan) showed the left eye of patient IV: 1. **(A)** The patient at 6 years of age (with a disease history of 4 years). The cornea was gray and opaque under the epithelium. However, the cornea in the pupil area still showed a lens presentation. **(B)** The patient at 12 years of age (with a disease history of 10 years). The corneal subepithelial gray opacity was thicker than in prior examinations and gradually fused into a map shape, blocking part of the pupil. **(C)** The patient at 15 years of age (with a disease history of 13 years). The entire cornea was seen as a gray and white opacification under the epithelium. **(D)** The patient was 17 years of age (with a disease history of 15 years). The disease had recurred 2 years after PTK. A white thin layer opacity of the cornea was observed and the range and shape of the opacity were the same as in the initial case presentation.

Table 1 showed the changes in the main clinical parameters for the six evaluated children presenting with CD. Avelino DNA test results showed that all affected children had an R124L heterozygous gene mutation. The average age of onset for the evaluated children with CD was 0.90 ± 0.61 years (0.25–2 years). The clinical manifestations were recurrent photophobia, tearing, and a foreign body sensation. Episodes occurred once every 2 months on average prior to 2 years of age. The incidence frequency gradually decreased with increasing age. Slit lamp examination revealed a rough corneal epithelium, as well as scattered grayish white turbidity under the corneal epithelium and the Bowman’s layer. The turbidity gradually fused into maps with age. From onset to follow-up, visual acuity decreased from 0.34 ± 0.12 to 0.55 ± 0.17 logarithm of the minimum angle of resolution (LogMAR) units (Figure 2). One pediatric patient received binocular PTK treatment and relapsed 1 year after surgery (Table 1).

**TABLE 1:** Clinical data of children with R124L mutation CD.

Position in the family	Gender	Age (years)	Age of onset (years)	History (years)	Time of first operation (years)	Symptoms				UDVA (LogMAR)		Operation	Postoperative recurrence
			
						Vision loss	Photophobia	Tears	Foreign body sensation	OD	OS		
IV: 1	Female	17	2	15	15	+	+	+	+	0.8	0.5	PTK (double)	Recurrence occurred 1 year after surgery
IV: 2	Female	2	0.25	1.75	–	+	+	+	+	*	*	–	–
IV: 3	Female	15	0.58	14.42	–	+	+	+	+	0.5	0.7	–	–
IV: 4	Male	7	0.58	6.42	–	+	+	+	+	0.5	0.4	–	–
IV: 5	Male	10	1	9	–	+	+	+	+	0.2	0.2	–	–
IV: 19	Female	3	1	2	–	+	+	+	+	*	*	–	–

“–”indicated that no surgery had been performed; “+” indicated the presence of positive symptoms; “*” indicated that the vision examination did not comply.

### Anterior segment optical coherence tomography and in vivo confocal microscopy examination

**FIGURE 3:**
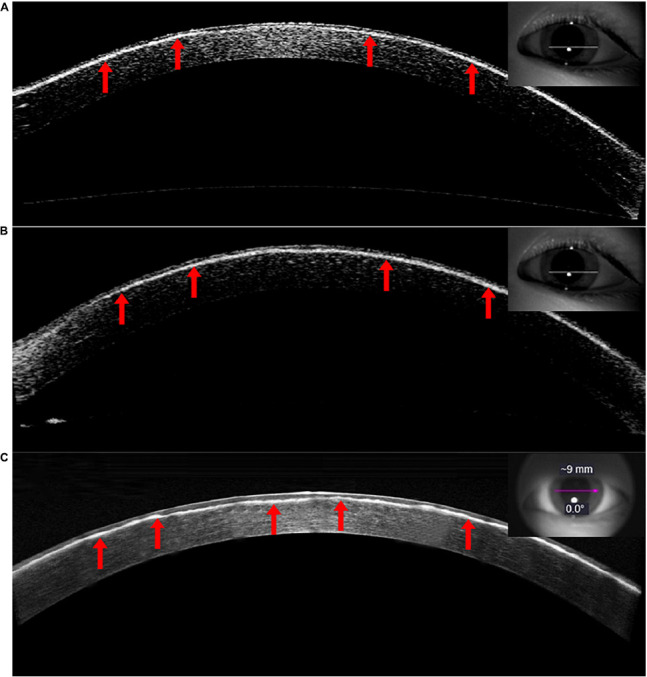
A depiction of the AS-OCT (Carl Zeiss Meditec, Jena, Germany) imaging of the left eye (VI:1). Patient **(A)** was 12 years of age with a medical history of 10 years. Uneven thickness of the corneal epithelium was observed. The Bowmanen layer had disappeared and the anterior corneal stroma was highly reflective, with an overall irregular morphology and multiple highly reflective ridges (Visante). Patient **(B)** was 15 years of age with a disease history of 13 years. The interlamellar highly reflective material increased gradually over time (Visante). Patient **(C)** was 17 years of age with a disease history of 15 years and recurrence 2 years after PTK. The corneal interlaminar deposits were similar to those in the initial case. Superficial corneal stroma ridges were more obvious in this presentation (HD-OCT).

AS-OCT revealed irregular corneal morphology in all children with CD, uneven thickness of the corneal epithelium, and disappearance of the anterior elastic layer. There was a high level of reflectivity in the subepithelial and anterior stroma of the cornea. Multiple highly reflective patches and ridges were also observed between the subepithelial and anterior stroma of the cornea. The thickness of the interlaminar corneal sediments was 102.78 ± 10.13 μm on average as of the last follow-up. The corneal interlaminar deposits of the patients with recurrence occurring following PTK were similar to those of patients at their initial presentation. However, corneal subepithelial hill-like ridges were more obvious in the disease presentation following PTK (Figure 3).

**FIGURE 4:**
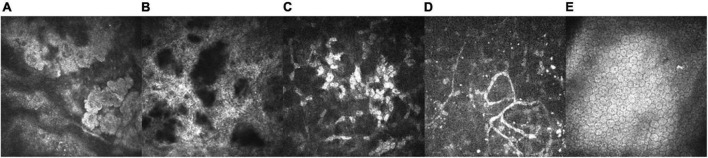
IVCM of the left eye in a 15-year-old patient with a 13 year disease history (VI: 1; X800, HRT-III, Heidelberg Engineering GmbH, Heidelberg, Germany). **(A)** We observed a large number of patchy uneven and highly reflective corneal epithelium patches with unclear boundaries. Epithelial cells lost their normal cell morphology and the cell space widened (25 μm). **(B)** The Bowmaner of patchy uneven and highly reflective corneal epithelium patches with unclear unrecognizable (73 μm). **(C)** A depiction of the anterior corneal stroma, presenting with high reflectivity and an unclear boundary (156 μm). **(D)** A small number of highly reflective signals and nerve fibers (329 μm) were observed in the deep corneal stroma. **(E)** The corneal endothelial cells were not abnormal upon examination (550 μm).

As the disease progressed, IVCM revealed many uneven patches of highly reflective light in the upper corneal cortex, with unclear boundaries. Epithelial cells lost their normal cell morphology and the cell space widened. The Bowman’s layer was completely replaced by a highly reflective material, such that the structure was unrecognizable. The anterior corneal stroma was likewise highly reflective and the boundary was unclear. A small number of highly reflective signals and nerve fibers were observed in the deep corneal stroma. No obvious abnormalities were observed in the corneal endothelial cells (Figure 4).

## Discussion

Recently, the development of novel and effective ophthalmic technology and equipment, such as AS-OCT and IVCM, have provided new opportunities for obtaining accurate and effective CD diagnoses. The histological characteristics of patients’ lesions can be thoroughly and comprehensively identified *via* AS-OCT and IVCM (4). In this study, AS-OCT and IVCM were used to observe the characteristics of CD as occurring in children in order to provide evidence for early clinical screening and monitoring of the disease course.

CD caused by an R124L mutation is an autosomal dominant anterior form of CD, which usually manifests as recurrent photophobia and tearing accompanied by vision loss. Slit-lamp examination typically shows erosion and shedding of the corneal epithelium in the early stages of the disease. A spot-like turbidity appears as the disease progresses. Later, turbidity fusion reveals map changes (5). In this study, the onset of CD caused by an R124L mutation generally occurred early in life (prior to 2 years of age). In the standard disease course, corneal epithelial erosion and detachment often occur repeatedly starting in childhood, with increasingly obvious symptomology and a rapid progression. Later in the disease course, corneal opacity gradually expands and fuses into a map, leading to vision loss and potentially leading to blindness. The youngest age of onset in the children enrolled in this study was 3 months. However, the age of onset was reported to be more than 3 years in prior studies (6). In the evaluated family, the reoccurrence of disease episodes in the evaluated children was approximately yearly or twice a year. Symptoms included red eyes, eye grinding, photophobia, tearing, and other uncomfortable symptoms. With increasing age, the onset and recurrence CD gradually decreases. In this study, one child was treated with PTK in both eyes. This child relapsed 1 year after surgery, and the time to relapse was earlier than that reported in prior work (3 years) (7).

In this study, the R124L locus mutation was replaced by the light of non-polar hydrophobic amino acids that meaningfully contribute to the three-dimensional structure of the protein due to the polarity of the essence of hydrophilic amino acids, thus leading to earlier visual impairment and rapid illness progression in this form of CD. Our work and that of other researchers and clinicians had established that CD easily relapsed after PTK or keratoplasty. However, recurrent CD could be successfully treated with PTK or keratoplasty (6).

Children with the R124L mutation generally show phased and progressive CD development. In the early stages (<9 years), multiple fine spot-like turbidities are observed in the corneal epithelium and the anterior stroma. In the long-term course of the disease (>14 years), the number and density of fine spot-like turbidities gradually increase. However, the density of corneal opacities is generally higher in adults than in children (6). The Bowman’s layer and anterior corneal stroma tend to gradually increase in thickness with increasing age.

In recent years, OCT has been widely used in clinical ophthalmology for the detection and screening of macular lesions, choroid-related diseases, and various corneal diseases (8). In the corneal plane, AS-OCT can accurately evaluate the overall structure of the corneal epithelium and stroma and is widely used in the diagnosis and treatment of a range of corneal diseases (9). In normal eyes, corneal tissue can be divided into three layers using AS-OCT. The superficial layers of the corneal tissue are the corneal epithelium and Bowman’s layer. The innermost highly reflective layers are the endothelial cell layer and the cement membrane. The low-reflective tissue between the two layers is termed the corneal stroma (10).

Patients with CD often present with an uneven distribution of the corneal epithelium and an abnormal interlayer deposition of corneal substances. In this study, AS-OCT showed good penetration and could clearly determine the location and depth of the lesion. To the best of our knowledge, AS-OCT findings in children with CD have not yet been reported to date. In this study, the corneal morphology of children with CD was irregular and the thickness of the corneal epithelium was uneven on AS-OCT. Highly reflective deposition was observed in the subepithelial and anterior corneal stroma. Sedimentation was found in the middle and mid-week areas of the cornea. The sediment front (i.e., toward the upper layer) was serrated with several highly reflective hill-like local ridges. The posterior margin of the sediment (toward the stroma) was well bounded.

With prolongation of the disease course as evaluated in the current study, the highly reflective material gradually became thicker. No highly reflective substances were detected in the deep cornea and no obvious abnormalities were observed in the corneal endothelium. The corneal morphology of the children with CD episode recurring after PTK was the same as that of the initial case presentations, with highly reflective substances observed in the subepithelial and anterior stroma of the cornea. However, we observed a more irregular corneal morphology and more obvious local hill-like ridges in those treated with PTK. It had been previously reported that AS-OCT findings in adults with CD induced by an R124L mutation showed highly reflective material in the Bowmand layer and in the anterior stroma. In five patients with a total corneal thickness of 566 ± 21 μm, the thickness of the highly reflective material in the Bowman layer and anterior stroma was 96 ± 12 μm (72–132 μm) (7). In this study, the six patients average thickness of the sediment was 102.78 ± 10.13 μm (80–117 μm), which was consistent with previous reports.

IVCM is a novel optical microscopy technique that can be used to analyze corneal tissue structure at the cellular level in vivo and in real-time. It offers the advantages of high magnification, high resolution, no resulting damage or adverse effects, and excellent repeatability. In recent years, it has also been applied in clinical diagnostics and research specific to CD (11). In normal corneal tissue, IVCM can clearly distinguish the three types of cells in the corneal epithelium (squamous, wing, and basal epithelium) (12). Dendritic cells can clearly be seen near the basement membrane of the corneal nerve fiber bundle. The density of the substrate layer of stromal cells and the collagen fiber mesh structure changes from shallow to deep. The endothelial cell boundary is clear and can be accurately evaluated *via* a count analysis (13).

In CD cases, IVCM can be used to observe the morphological changes of various cell components as well as corneal precipitation, which has important clinical significance in the accurate diagnosis of CD (14). In this study, IVCM implemented in children with CD showed that the evaluated corneal epithelial cells lost their normal structure, with many uneven and highly reflective signals. The Bowmanis study was replaced by highly reflective substances, and the tissue structure could not be observed. The anterior stroma was highly reflective with unclear boundaries. No obvious abnormalities were observed in the deep stroma or in the corneal endothelial cells. Conversely, previous studies have reported that IVCM examinations in adult patients with CD revealed an intact corneal epithelium. Moreover, in these examinations, the Bowmanved in thead disappeared. The corneal subepithelial nerve, where sediment was located, had likewise disappeared. However, sediment was found in the anterior stroma. Only a few nerves or short nerve tissues were found in the posterior stroma (6). As a contact operation examination, IVCM requires a high degree of cooperation from children. Therefore, one 2-year-old patient with CD was extremely uncooperative and ultimately failed to complete the examination. The current study only performed this examination on the other five older children who were capable of providing a high degree of cooperation.

Compared with the adults with CD, CD in children had earlier onset, faster progression, and longer disease course. In addition, children could not express themselves accurately, early diagnosis and treatment could be difficult. Based on our study, AS-OCT combined with IVCM could be used to perform a real-time three-dimensional analysis of the corneal tissue structure in vivo to determine the characteristics and depth of lesions in children with CD. However, the resolution of AS-OCT is lower than that of IVCM. AS-OCT can clearly determine the structure, size, and depth of the corneal tissue. Moreover, AS-OCT has the advantages of a convenient and quick examination and a lack of contact and invasiveness, and likewise allows for a greater degree of intracorneal analysis. The purpose of this study was to observe the characteristics of CD in children and aimed to provide guidance for early clinical diagnosis. AS-OCT as a practical and effective tool for the early screening and monitoring of the pathological process in CD, it is worthy of clinical application.

This study had some limitations. For example, we only studied one family with a history of incest/consanguineous marriage. Examinations enrolling more families could lead to more reliable and conclusive findings.

## Conclusion

In conclusion, in this family with prevalent CD caused by an R124L mutation, we reported an early age of onset in the affected children. AS-OCT and IVCM examinations demonstrated an abnormal accumulation of subepithelial and anterior corneal stroma. Thus, we conclude that AS-OCT provides highly useful information regarding CD. This is a convenient and efficient tool with a lack of contact that is efficacious in the early screening and monitoring of the pathological processes occurring in CD and we can confidently conclude that it is worthy of clinical application. However, our findings would be strengthened by research conducted in more highly powered investigations.

## Data availability statement

The original contributions presented in this study are included in the article/supplementary material, further inquiries can be directed to the corresponding author/s.

## Ethics statement

The studies involving human participants were reviewed and approved by the Weifang Eye Hospital Review Ethics Committee. Written informed consent to participate in this study was provided by the participants’ legal guardian/next of kin.

## Author contributions

MF and JZ: conduct the study and writing the manuscript. MF, HZ, and RW: data collection. XZ: revising the manuscript. MF and XZ: designing the study. All authors contributed to the article and approved the submitted version.
